# How the brain produces generalized fear

**DOI:** 10.1002/ctm2.70124

**Published:** 2025-01-17

**Authors:** Hui‐Quan Li, Nicholas C. Spitzer

**Affiliations:** ^1^ Neurobiology Department and Kavli Institute for Brain and Mind University of California San Diego La Jolla California USA; ^2^ Neurocrine Biosciences Inc. San Diego California USA

1

Fear alerts us to threats and is essential to survival. Acquired fear that is associated with a specific stimulus is defined as conditioned fear. However, fear can frequently generalize to other stimuli and contexts, and this generalized fear to harmless situations is a key component of anxiety that can result from acute stress. Generalized fear that is inappropriate to the stimuli that provoke it can be disadvantageous, destructive and even dangerous. Understanding how fear generalization occurs and how it can be controlled may suggest directions for the development of novel therapies to treat or even cure fear disorders.

In our study, we investigated the effect of footshock, a form of acute stress, which causes freezing behaviour that is a measure of fear in rodents. We found that a mild footshock given to mice produced only conditioned fear, but a strong footshock produced both conditioned and generalized fear (Figure 1A). We also found that footshock produced conditioned fear immediately, but generalized fear was present only after a three‐day delay. The production of generalized fear was tightly associated with a change in co‐transmitter identity from excitatory neurotransmitter glutamate to inhibitory neurotransmitter gamma‐aminobutyric acid (GABA) in serotonergic neurons in the dorsal raphe (Figure 1B).^1^ No change in birth or death of neurons was detected that could account for changes in neurotransmitter expression.

**FIGURE 1:**
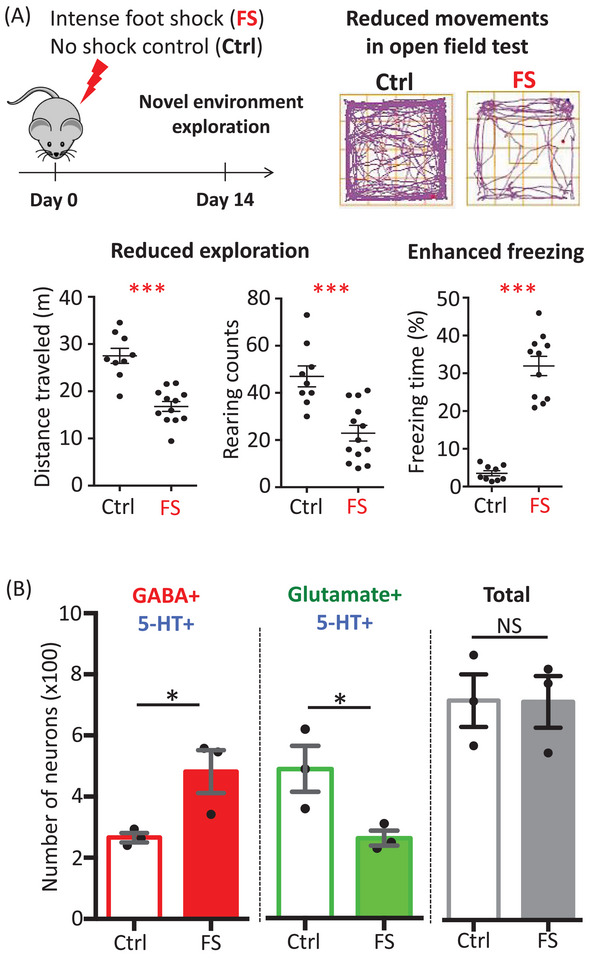
Footshock induces fear behaviour in a novel environment that depends on changes in co‐transmitter identity in neurons in the lateral wings of the dorsal raphe. (A) Footshock reduces movement in the open field test (distance travelled and rearing counts) and increases freezing behaviour. (B) Footshock increases the number of serotonergic neurons that co‐express GABA and reduces the number that co‐express glutamate.

Using a stable genetic marker to track the neurons, the change in co‐transmitter was seen to occur in single cells—thus revealing a co‐transmitter switch. There was no gender difference identified for the production of either conditioned or generalized fear or for the induction of the transmitter switch. The switching neurons made connections to neurons in the central amygdala and lateral hypothalamus, which are regions of the brain that mediate fear responses.

To learn whether the findings in rodents were translatable to humans, we then examined the postmortem brains of individuals with and without posttraumatic stress disorder (PTSD) provided by the National Institutes of Health NeuroBioBank. We observed a change in co‐transmitter expression in the brains of individuals with PTSD, but not in the brains of age‐, gender‐, and postmortem interval‐matched control subjects. The changes seen in PTSD individuals are consistent with those observed in footshocked mice that exhibited generalized fear.

When we suppressed the synthesis of GABA in footshock mice using adeno‐associated virus (AAV)‐based gene transfer tools to interfere with the expression of GABA synthase, we prevented the appearance of generalized fear in response to footshock. This result suggested that the transmitter switch is necessary for the acquisition of generalized fear. Re‐establishing the function of glutamatergic transmission, by restoring the lost glutamate transporters using AAV tools, was not as effective as suppressing the expression of GABA in blocking the appearance of generalized fear. Overall, generalized and conditioned fear differed in induction threshold to shock,^1^, ^2^ in time course,^1^, ^3^ and in dependence on the glutamate‐to‐GABA switch in the serotonergic neurons.^1^

Footshock also activates the stress pathway and causes the release of the stress hormone, corticosterone (a rodent equivalent to cortisol in humans). Pharmacological suppression of corticosterone release or blockage of glucocorticoid receptors prevented transmitter switching and the appearance of generalized fear. The application of corticosterone without footshock was insufficient to drive the expression of generalized fear, but the application of corticosterone together with a mild shock was sufficient to drive the co‐transmitter switch and trigger generalized fear. These results are consistent with observations that injection of corticosterone increases GABA release and reduces glutamate release in the adult rodent amygdala^4^ and upregulates GAD67 messenger RNA in the hippocampus.^5^ In addition to blocking corticosterone synthesis, we found that either blocking the neuronal activity of corticosterone‐releasing hormone neurons in the paraventricular nucleus or knocking down the glucocorticoid receptor in the dorsal raphe also blocked the co‐transmitter switch in the serotonergic neurons.

We then tested the effect of fluoxetine, a selective serotonin reuptake inhibitor (SSRI) antidepressant drug, on the acquisition of generalized fear. Fluoxetine has been shown to reduce stress‐induced conditioned fear. Following footshock, prompt treatment with fluoxetine prevented the co‐transmitter switch and prevented the appearance of generalized fear (Figure 2). Chronic treatment with antidepressants inhibits glucocorticoid receptor‐mediated gene transcription^6^, ^7^ and reverses corticosterone‐induced anxiety/depression‐like behaviour.^8^ Thus, prompt fluoxetine may block the co‐transmitter switch in the dorsal raphe by blocking glucocorticoid receptor‐mediated gene transcription in serotonergic neurons. The mechanisms by which prompt fluoxetine prevents the co‐transmitter switch require further investigation.

**FIGURE 2:**
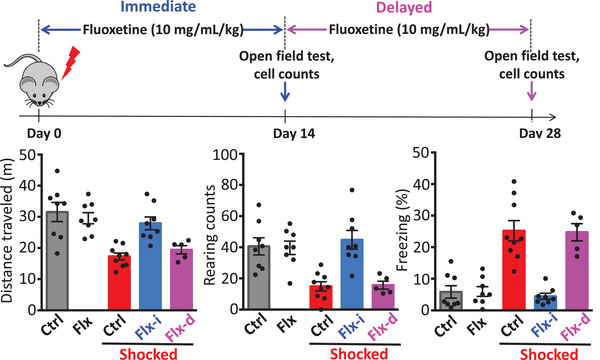
Prompt treatment with Fluoxetine prevents the appearance of generalized fear. Immediate treatment following footshock prevents reduction in exploratory behaviour (distance travelled and rearing counts) and prevents increased freezing. When treatment is delayed until day 14 it is no longer effective.

However, delayed treatment with this drug after two weeks prevented neither the switch nor the generalized fear. The short time window for effective therapy may explain why PTSD patients with long histories of this disorder are less responsive to SSRIs.^9^ Considering the present investigation and a previous study,^10^ the dorsal raphe appears to be a hot spot for neurotransmitter switching that is relevant to physiological disorders. The dynamics of neurotransmitters in subregions of the dorsal raphe may deserve further investigation 1 and 2.

Our findings provide insight into the changes in the brain produced by acute stress, which give rise to generalized fear often referred to as PTSD. Following acute stress, prompt delivery of fluoxetine can be a beneficial treatment. Transmitter switching in neurons has also been linked to behavioural changes such as improvements of motor function,^11^, ^12^, ^13^ induction of depression by altered photoperiod^14^, ^15^ induction of autism spectrum disorder by environmental stimuli,^16^ cognitive deficits produced by drugs of abuse^17^, ^18^ and therapies for Parkinson's disease.^19^, ^20^ The occurrence and roles of neurotransmitter switching in many brain regions remain to be explored. Cutting‐edge methodologies such as single‐cell RNAseq or whole‐brain scale imaging techniques will enable faster and deeper exploration of the roles of neurotransmitter switching, and provide further insights into neurological and psychiatric disorders.^21^

## CONFLICT OF INTEREST STATEMENT

The authors declare no conflict of interest.
